# Early Morning QT Prolongation During Hypoglycemia: Only a Matter of Glucose?

**DOI:** 10.3389/fcvm.2021.688875

**Published:** 2021-05-11

**Authors:** Sara D'Imperio, Michelle M. Monasky, Emanuele Micaglio, Gabriele Negro, Carlo Pappone

**Affiliations:** ^1^Arrhythmology Department, IRCCS Policlinico San Donato, Milan, Italy; ^2^Vita-Salute San Raffaele University, Milan, Italy

**Keywords:** QT prolongation, hypoglycemia, Brugada syndrome, diabetes, sudden cardiac death, glucose, glucose - insulin

QT is the surface ECG equivalent of the time it takes for the ventricular myocardium to repolarize after its depolarization. The prolongation of the QT interval is a dangerous ECG abnormality that can be inherited or acquired and is a risk for sudden cardiovascular events due to its potential arrhythmogenicity. The prevalence of QT prolongation has been investigated in both type 1 and type 2 diabetes mellitus (DM) (1). It has been demonstrated that QT prolongation is a risk factor for sudden cardiac death in the presence of hypoglycemia (2), including when it is insulin-induced (3).

We read with great interest the retrospective study by Tsujimoto et al. reporting that in the early morning (4 a.m.−10 a.m.) severe hypoglycemia was associated with a significant incidence of abnormal QT prolongation in a cohort of 287 Japanese patients, regardless of whether the patients were diabetic or not (4). This study focused on the relationship between QT prolongation and severe hypoglycemia, which is a common and deleterious effect of diabetes and has been associated with adverse cardiovascular outcomes, such as fatal cardiac arrhythmic events (5, 6) and mortality (7). Indeed, several studies support that there is a strict correlation between hypoglycemia and arrhythmic events during bedtime in diabetic patients (6, 8–10), with a significant increase in prolonged QT interval (11–13). Of note, in the human species, the length of the QT interval has been shown to depend upon the circadian rhythm, which is controlled by the autonomous nervous system and was shown to have marked variability in patients with type 1 diabetes mellitus (14). We are aware that maturity onset diabetes of the young (MODY) is not a common cause of diabetes worldwide, although an excellent study by Yorifuji et al. (15) reported 103 mutations in MODY genes (39%) in a cohort of 263 Japanese diabetic patients. Therefore, we suggest the analysis of at least the genes *GCK, HNF1A, HNF1B*, and *HNF4A* in the cohort presented by Tsujimoto and coworkers.

Lengthened QT interval is associated with ventricular arrhythmias and sudden cardiac death (16). It can be caused by genetic variations, mineral imbalance, or QT-prolonging medications (17, 18). Moreover, a higher incidence of QT prolongation has been described in metabolic syndrome (19), eating disorders, such as anorexia nervosa (20), and obesity (21). Therefore, it is very important to identify the underlying conditions of the patients that influence their QT interval and risk of mortality.

Multiple regulatory mechanisms are implicated in balancing the serum glucose concentration with the circadian rhythm. Therefore, when this balance is disrupted, hypoglycemia can occur. This condition usually occurs in diabetic patients due to the administration of hypoglycemic agents, such as insulin or sulfonylurea. However, hypoglycemia can develop due to several clinical complications, such as metabolic diseases (22), insulinoma (23), and non-islet tumors (24). Moreover, regardless of a patient's diet, hypoglycemia can also manifest due to an excess intake of alcohol, because it inhibits both hepatic gluconeogenesis and glycogenolysis (25–27). Autoimmunity is another important cause of both hypoglycemia and QT interval prolongation. Type 1 diabetes is characterized by the presence of many different antibodies (28). Those antibodies provoke autoimmune destruction of insulin-producing β cells (29) and can be detected in the patients' plasma. Thus, it is very important to exclude the presence of such mechanisms in the studied cohort.

Of note, in the present article by Tsujimoto et al., we did not find any information regarding the clinical status of non-DM patients, in particular the prevalence of potentially QT prolonging conditions [hypokalemia, QT prolonging drugs, metabolic disorders others than diabetes, or inflammation (30)]. Therefore, it would be better to understand the etiology of the severe hypoglycemia, because it may be the cause or a contributing cause of the QT prolongation. This ECG abnormality happens spontaneously in the hereditary form, but can be triggered by drugs (https://www.crediblemeds.org), such as antiarrhythmics, antihistamines, antipsychotics, antibiotics (18), cancer treatments, and alcohol intake (25, 26). Furthermore, no genetic testing is presented about the most common long QT syndrome (LQTS) genes (*KCNQ1, KCNH2, SCN5A*, or possible modulating polymorphisms).

Moreover, we noticed that Tsujimoto et al. only took into consideration the QT interval and no other important electrocardiogram (ECG) information, such as ST-segment and T wave. Clinical similarities exist among patients with prolonged QT interval and Brugada syndrome (BrS), and the majority of these patients are asymptomatic, yet they can be at risk of sudden cardiac death (31–33).

BrS and LQTS are both life-threatening inherited arrhythmic disorders that usually manifest in the nighttime. LQTS type-3 and BrS also share the same *SCN5A* variant (34, 35), and several instances of an overlap syndrome have been described (34, 36). Therefore, the evaluation of the ST-segment could have been useful to unveil an overlap syndrome.

We understand why cardiopulmonary arrest patients were excluded from the study, however, it would have been useful to evaluate, if available, the ECGs of those patients, since they showed the worst phenotype. Therefore, it would have been interesting if those patients had prior clinical evaluations of their health conditions. Specifically, it would have been useful to know if they have ever had any symptoms like syncope, palpitations, seizures, or family history related to BrS, and more importantly, whether the ST-segment from a standard 12-precordial lead ECG had ever been evaluated. Notably, the patients presented to a hospital in Japan, which has a relatively high incidence of BrS, ranging from 0.1 to 0.2% in the general population (37).

It is known that many cardiovascular outcomes vary in prevalence depending the time of the day. Indeed, the cardiac circadian rhythm is involved in different cardiac functions and it may contribute to HF and SCD (38). We noticed that in the study conducted by Tsujimoto et al., the QT prolongation was only assessed in patients with severe hypoglycemia. It would have been helpful to have QT prolongation records of the same patients or a matched cohort, during periods of non-hypoglycemia. Moreover, to better understand the relationship between QT prolongation and hypoglycemia events, it would also be important to know the QT baseline at different times of day in order to determine the effect of the circadian fluctuations on the QT interval. In fact, two types of circadian clocks exist: a central clock that acts on the heart by various neurohumoral factors, and a local clock in the heart that alters the expression of cardiac ion channels (39). At night, the QT interval (as well as the PR interval and QRS duration) is lengthened, indicating slower ventricular repolarization. A possible mechanism for this may include ion channel remodeling, including Na^+^, Ca^2+^, and K^+^ channels, as well as connexins, as a result of circadian rhythm, as demonstrated in animal models (39). Therefore, in the study by Tsujimoto et al., it is not clear how it is possible to distinguish the role of hypoglycemia vs. the role of the time of day impacting the QT when the study included only data investigating hypoglycemia during early morning.

On another note, only patients with hypoglycemia were included in the study, but the relationship between blood glucose and QT prolongation was not discussed. A strict temporal relationship has been described between hypoglycemia and QT prolongation, stressing the proarrhythmic harm related to dynamic changes of heart repolarization (40). Thus, it would have been optimal to understand the duration of QT prolongation at different blood glucose concentrations. Indeed, this issue would have been better understood if several blood glucose values, including non-hypoglycemic readings, could have been included.

In conclusion, according to current literature, it is not possible to consider an abnormal QT prolongation during the early morning as just an expression of severe hypoglycemia. In fact, QT trait prolongation can be an expression of a concealed channelopathy (possibly overlap syndrome), a sign of non-alcoholic fatty liver disease, regardless of the presence of diabetes (41), or the onset of metabolic dysregulation, potentially associated with several different cardiomyopathies with preserved ejection fraction (HFpEF) (42).

## Author Contributions

SD'I and MM decided on the project, drafted the original version, and revised the manuscript. EM and GN provided expertise and revised the manuscript. CP provided resources and funding. All authors contributed to the article and approved the submitted version.

## Conflict of Interest

The authors declare that the research was conducted in the absence of any commercial or financial relationships that could be construed as a potential conflict of interest.
